# Machine learning-based identification of a novel prognosis-related long noncoding RNA signature for gastric cancer

**DOI:** 10.3389/fcell.2022.1017767

**Published:** 2022-11-11

**Authors:** Linli Zhao, Qiong Teng, Yuan Liu, Hao Chen, Wei Chong, Fengying Du, Kun Xiao, Yaodong Sang, Chenghao Ma, Jian Cui, Liang Shang, Ronghua Zhang

**Affiliations:** ^1^ Department of Ultrasound, Qilu Hospital of Shandong University, Jinan, Shandong, China; ^2^ Department of Gastrointestinal Surgery, Shandong Provincial Hospital, Shandong University, Jinan, Shandong, China; ^3^ Clinical Epidemiology Unit, Clinical Research Center of Shandong University, Qilu Hospital of Shandong University, Jinan, Shandong, China; ^4^ Department of Gastrointestinal Surgery, Shandong Provincial Hospital Affiliated to Shandong First Medical University, Jinan, Shandong, China; ^5^ Key Laboratory of Engineering of Shandong Province, Shandong Provincial Hospital, Jinan, Shandong, China; ^6^ Medical Science and Technology Innovation Center, Shandong First Medical University and Shandong Academy of Medical Sciences, Jinan, Shandong, China; ^7^ BioGeniusCloud, Shanghai BioGenius Biotechnology Center, Shanghai, China

**Keywords:** gastric cancer, long noncoding RNA, prognostic signature, immunotherapy, machine learning algorithm

## Abstract

Gastric cancer (GC) is one of the most common malignancies with a poor prognosis. Immunotherapy has attracted much attention as a treatment for a wide range of cancers, including GC. However, not all patients respond to immunotherapy. New models are urgently needed to accurately predict the prognosis and the efficacy of immunotherapy in patients with GC. Long noncoding RNAs (lncRNAs) play crucial roles in the occurrence and progression of cancers. Recent studies have identified a variety of prognosis-related lncRNA signatures in multiple cancers. However, these studies have some limitations. In the present study, we developed an integrative analysis to screen risk prediction models using various feature selection methods, such as univariate and multivariate Cox regression, least absolute shrinkage and selection operator (LASSO), stepwise selection techniques, subset selection, and a combination of the aforementioned methods. We constructed a 9-lncRNA signature for predicting the prognosis of GC patients in The Cancer Genome Atlas (TCGA) cohort using a machine learning algorithm. After obtaining a risk model from the training cohort, we further validated the model for predicting the prognosis in the test cohort, the entire dataset and two external GEO datasets. Then we explored the roles of the risk model in predicting immune cell infiltration, immunotherapeutic responses and genomic mutations. The results revealed that this risk model held promise for predicting the prognostic outcomes and immunotherapeutic responses of GC patients. Our findings provide ideas for integrating multiple screening methods for risk modeling through machine learning algorithms.

## Introduction

Gastric cancer (GC) is the third leading cause of cancer-related deaths worldwide and has a poor prognosis (Siegel et al., 2022). Due to changes in dietary composition, work pressure, *Helicobacter pylori* infection and other factors, the incidence of GC is increasing and, and it is increasingly being detected in younger individuals (Chandra et al., 2021). Systemic chemotherapy, surgery, immunotherapy, and targeted therapy have proven efficacy against GC (Joshi and Badgwell, 2021). With the advancement of technology, some progress has been achieved in the diagnosis and treatment strategies for GC, but there was no significant improvement in overall survival, particularly for patients with advanced GC. Recent advances in immunotherapy have attracted considerable attention to its use as a viable therapeutic option for several cancers. Numerous studies have shown that immunotherapy might significantly prolong both overall survival (OS) and progression-free survival (PFS) in patients with GC (Ajani et al., 2022). However, not all GC patients are sensitive to immunotherapy (Kole et al., 2022). Therefore, novel prognostic and diagnostic markers or models are urgently needed to accurately predict the efficacy of immunotherapy in patients with GC.

Emerging evidence has indicated that the specific tumor microenvironment (TME) in GC tissues plays vital roles in the occurrence and development of GC (Kono et al., 2020). The TME is a complicated system, comprising a variety of cellular components, including immune cells, fibroblasts, nerve cells, and vascular endothelial cells. Cross-talk between cancer cells and stromal cells in the TME ultimately shapes an environment that promotes tumor growth and metastasis. (Hinshaw and Shevde, 2019; Oya et al., 2020). GC cells recruit tumor-associated macrophages (TAMs), myeloid derived suppressor cells (MDSCs) and regulatory T cells (Tregs) by secreting cytokines and chemokines to inhibit the T lymphocyte immune response and promote the generation of an immunosuppressive state in GC (Jiang et al., 2019; Derks et al., 2020). Tregs have been shown to promote tumor escape from cytotoxic immune responses, leading to the inactivation of tumor immune effector cells. The accumulation of MDSCs in GC is associated with immune checkpoint inhibitor resistance, and by reducing the accumulation of MDSCs, the infiltration of CD8^+^ T cells in GC might be increased, and further enhancing anti-PD-1 antitumor efficacy (Zhou et al., 2022). Cells in the TME activate and stimulate other cell types, creating an immunosuppressive status that promotes tumor cells to escape from immune surveillance and become resistant to tumor therapy (Oya et al., 2020). Thus, further investigations of cellular interactions in the TME are essential for the development of novel cancer therapies.

Long noncoding RNAs (lncRNAs) are a group of noncoding transcripts longer than 200 nucleotides, that play crucial roles in the occurrence and progression of cancers (Bhan et al., 2017). LncRNAs have been proven to be related to cancer development and immunity, and they have attracted increasing attention as biomarkers for early diagnosis and treatment, and for assessing drug resistance in GC patients (Yuan et al., 2020). LncRNAs can regulate the expression of genes related to the immune response and change the state of immune cells in the TME, thus affecting the aggressiveness, progression and prognosis of cancers. Therefore, further study of the relationship between prognosis-related lncRNAs and cancer immunity may help improve patient prognosis and identify novel therapeutic targets. By analyzing public databases, recent studies have identified many prognosis-related lncRNA signatures for cancers, including GC (Liu et al., 2020; Ma et al., 2021; Huang et al., 2022a). However, due to the particularity of The Cancer Genome Atlas (TCGA) and other databases, these studies did not standardize the clinical data obtained from the databases. For example, some tumor node metastasis (TNM) staging in some papers was performed using very old versions of the standards, and patients with distant metastases were not excluded from the analysis. Therefore, clinical data must be preprocessed and multiple methods should be integrated to obtain an accurate and effective prognostic model.

In this study, we performed a machine learning-based integrative analysis using public TCGA datasets and randomly divided all GC patients into training and test cohorts in a 1:1 ratio after the normalization and standardization of the clinical data. We manipulated the signature using various feature selection methods, such as univariate and multivariate Cox regression, least absolute shrinkage and selection operator (LASSO), stepwise selection techniques, subset selection, and a combination of above methods. We generated a 9-lncRNA signature for predicting the prognosis of patients with GC using a machine learning algorithm and explored the roles of the risk model in predicting immune cell infiltration, immunotherapeutic responses and genomic mutations. Our results indicated that this risk model might be promising for predicting the prognostic outcomes and immunotherapeutic responses of GC patients.

## Materials and methods

### Data collection and preprocessing from public databases

Using datasets from TCGA and the Gene Expression Omnibus (GEO), the differentially expressed genes (DEGs) between cancer tissues and adjacent normal tissues were identified. After careful filtering, only the TCGA dataset containing both mRNA and lncRNA expression data was considered. The RNA-sequencing data obtained from GC patients were retrieved from TCGA database (https://gdc.cancer.gov/). The corresponding clinical information was downloaded from the Genomic Data Commons (GDC) portal (https://portal.gdc.cancer.gov/) under the cohort named “TCGA Stomach Cancer (STAD)”. The Fragments Per Kilobase of transcript per Million mapped reads (FPKM) values from TCGA were log2 normalized. After the collection of TCGA clinical data from the GDC, 354 patients from TCGA database were first randomly divided into training and test cohorts at a ratio of 1:1 and patients with complete information on survival time and survival status were selected for further analysis. The TNM staging of the patients was standardized according to the eighth edition, while patients whose TNM staging was performed using the fourth edition were excluded since the staging criteria did not correspond with those in the eighth edition. Patients with distant metastases (M1), uncertain TNM staging (Tx/Nx), uncertain histologic grade (Gx) and a survival time of 0 were also excluded from the analysis. The stromal score, immune score and ESTIMATE score of each patient were calculated (Yoshihara et al., 2013). Since the data were downloaded from a publicly available database, the requirement for ethical approval was waived for this study.

### Establishment and validation of the risk model

The potential prognostic genes were screened using the LASSO algorithm (Dai et al., 2021), univariate and multivariate Cox regression analyses, stepwise selection techniques, subset selection techniques, and Kaplan-Meier analysis based on OS and a definition of significance of *p* values < 0.05. By automatically tuning the candidate prognosis-related signatures, we first designed a strategy, named TCGA Biomarkers, that used univariate Cox regression, LASSO, and multivariate Cox regression models to identify candidate prognostic signatures, and then conducted DeepSelector analysis, which took the common or union of the above various independent feature selection methods by controlling the signature size less than max size, e.g.,100, or greater than two (we also set default priority in sequential order when conflicting choices occurred). Then, we used the candidate signatures as input for the SubsetSelector (subset selection *via* BESS) (Ozhan et al., 2021) and StepwiseSelector (stepwise selection *via* stepAIC) (Jungk et al., 2019) engines and finally harmonized both filtered biomarkers from SubsetSelector and StepwiseSelector using a common or union strategy and finally obtained candidate biomarkers for training multivariate Cox regression analyses. During harmony processing, when conflicting conditions occurred, we also considered sequential selections of various methods, such as stepAIC as the first step, and BESS, and DeepSelector or singular selector afterward. We also designed a method named SumRank, which was used to calculate the voting frequency according to all other calculated methods (counting the frequency selected as a marker for each lncRNA, the selected number/total method number), and we then used the top 10% of the lncRNAs as the signature (Nematzadeh et al., 2019). The RobustRankAggreg package in R software was also used to identify the lncRNA signatures (Zhou et al., 2021). The potential prognostic lncRNAs identified using TCGA Biomarkers strategy pipeline were entered into the multivariate Cox regression analyses to identify a lncRNA signature in the training cohort. Univariate Cox regression analysis, multivariate Cox regression analysis, Kaplan-Meier survival curve, area under the curve (AUC) values obtained from the receiver operating characteristic (ROC) curves, Akaike’s information criterion (AIC) and concordance index (C-index) were used to filter the best risk model. The risk score of each patient was calculated using the formula: risk score=e^(∑i=1n(coefficient(gene i)*expression(gene i))). In this formula, “n”, “i”, and “coefficient” represent the number of selected lncRNAs, lncRNA numbers, and the multivariate Cox regression coefficients, respectively, and “expression” indicates the log_2_(FPKM+1) lncRNA expression level. Based on the optimal ROC curve, patients in the training cohort were divided into high- and low-risk groups. Decision curve analysis (DCA), calibration curves, time-dependent ROC curve analyses and Kaplan-Meier analysis were performed to evaluate the prognostic value of the lncRNA signature in the training cohort using the R packages “rmda”, “rms”, “survival”, “survivalROC”, and “survmier”. The expression levels of the nine lncRNAs in the risk model were visualized in a heatmap. The same formula and strategies were repeated in the test cohort and the merged cohort to assess the reliability of the lncRNA signature in predicting the prognosis.

### Clinical significance of the risk model

The relationship between the risk scores and clinicopathological characteristics of patients with GC in the three cohorts was determined using the chi-square test. Kaplan-Meier analysis was performed to determine the correlation between the risk model and prognosis of GC patients. Time-dependent ROC curves were constructed to test the accuracy of the risk model in both cohorts using the R packages “timeROC” and “survivalROC”. Univariate and multivariate analyses were performed to determine whether the risk score was associated with the prognosis of patients.

### Functional enrichment analysis

The DEGs identified between the high- and low-risk groups in the merged cohort were analyzed using the “limma” R package. DEGs with a fold change (FC) > 1.2 and a Benjamini–Hochberg adjusted *p* value < 0.05 were considered. Gene Ontology (GO), Kyoto Encyclopedia of Genes and Genomes (KEGG), Gene Set Enrichment Analysis (GSEA) and WikiPathways analyses were performed to investigate the functions and pathways of the DEGs. The GO and KEGG analyses were performed by using the “clusterProfiler” R package. The hallmark gene sets of GSEA were obtained from the Molecular Signatures Database (MSigDB v7.5.1, https://www.gsea-msigdb.org/gsea/msigdb/). A WikiPathways (https://www.wikipathways.org) analysis was performed as previously described to assess metabolism and signaling pathways (Martens et al., 2021).

### Evaluation of immune cell infiltration

We explored the relationship between the risk score, immune cell infiltration and immunotherapy efficacy by performing a quanTIseq (http://icbi.at/quantiseq) analysis, a method used to quantify the fractions of ten immune cell types from bulk RNA-sequencing data (Finotello et al., 2019). The difference in the levels of infiltrating immune cells between the high-risk and low-risk groups was visualized using the R packages “pheatmap”, “corrplot”, “ggpubr”, “ggplot2”, and “data.table”. The Tumor Immune Dysfunction and Exclusion (TIDE) algorithm was used as previously reported to further investigate the relationship between the risk score and T-cell dysfunction, (Jiang et al., 2018). We also explored the correlations between the risk score and the PD-L1 expression [log_2_ (TPM +1)].

### Tumor mutation burden

Tumor mutation burden (TMB) is a new biomarker currently under investigation. The TMB data obtained from GC patients were downloaded from the TCGA database (https://tcga-data.nci.nih.gov/tcga/). Using the “ggpubr”, “reshape2”, and “ggplot2” packages in R software, the relationship between the risk score and TMB was analyzed and visualized. The TMB statuses of patients in the high-risk group and low-risk group were obtained using the “maftools” package. Kaplan-Meier analysis was performed to analyze the differences in the survival of GC patients presenting with different TMB statuses and risk scores.

### Collection of clinical tissues and cell samples and quantitative real-time polymerase chain reaction

Sixteen pairs of GC tissues and corresponding adjacent nontumor tissues were collected from Shandong Provincial Hospital, which was approved by the Ethics Committee of the hospital. Cells from two human gastric cancer cell lines (MKN-45 and AGS) and one human gastric epithelial cell line (GES-1) were also collected. Total RNA was extracted from tissues or cells using TRIzol (Takara, Japan) according to the manufacturer’s protocol. Complementary DNA (cDNA) sequences were synthesized using a reverse transcription kit (Yugong Biolabs, Cat:EG15133S). All primers used for qRT-PCR designed and synthesized by GENEWIZ (Suzhou, China). The qRT-PCR was performed using Taq SYBR Green qPCR Premix (Yugong Biolabs, Cat:EG20117M) according to the manufacturer’s instructions. GAPDH was used as an internal control.

### Statistical analysis

The statistical analysis was conducted using R (version 4.1.0) software. The chi-square test and *t* test were applied to analyze the correlation between risk levels and clinicopathological characteristics, respectively. The Wilcoxon rank-sum test and Kruskal-Wallis test were used to evaluate the correlations of the risk score with clinical factors and other evaluated factors. Survival analyses were performed using the Kaplan-Meier method and the log-rank test using the package “survminer” in R. Results with a *p* value of <0.05 were considered statistically significant.

## Results

### Identification of a prognosis-related lncRNA signature for gastric cancer patients

**FIGURE 1:**
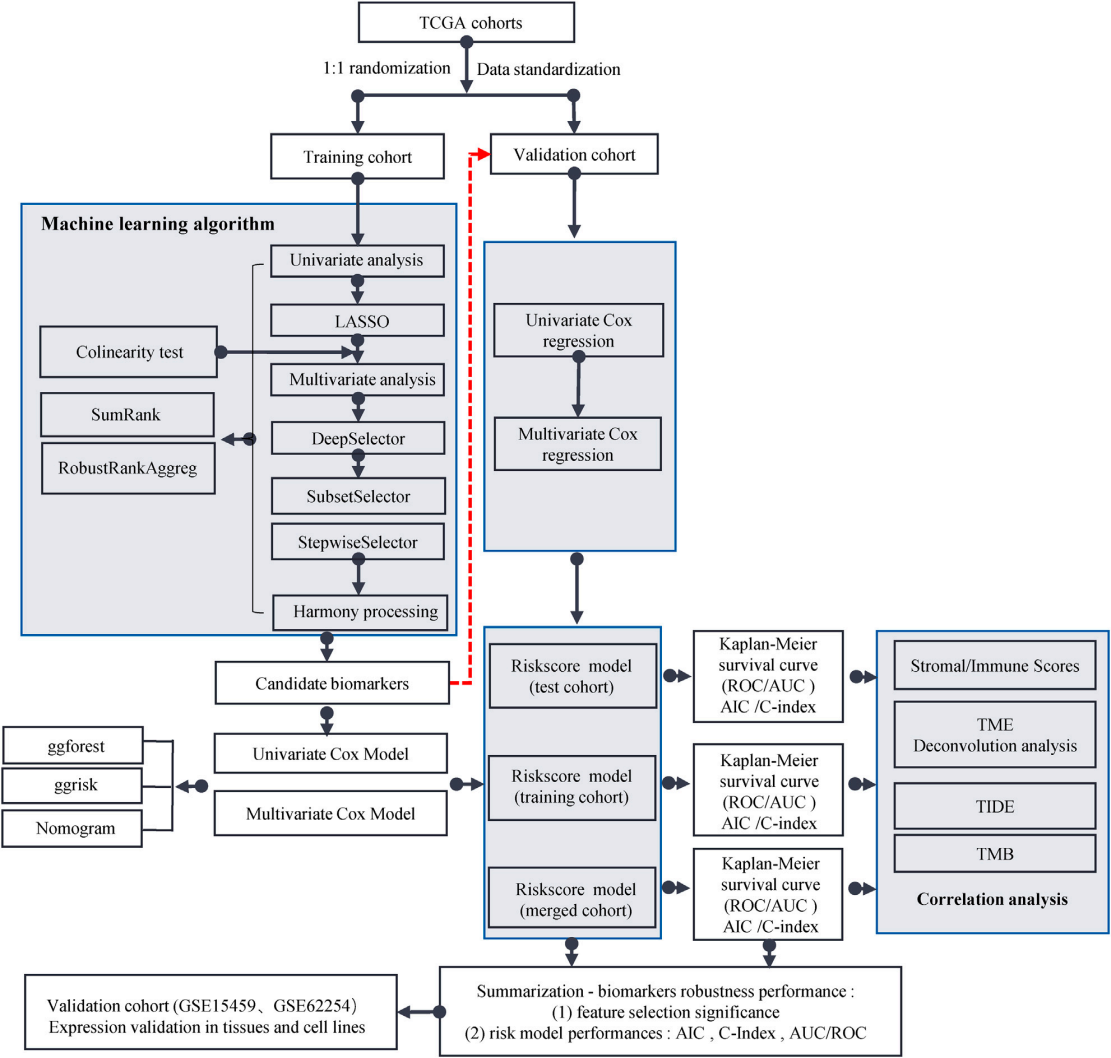
Flow chart of this study.

**FIGURE 2:**
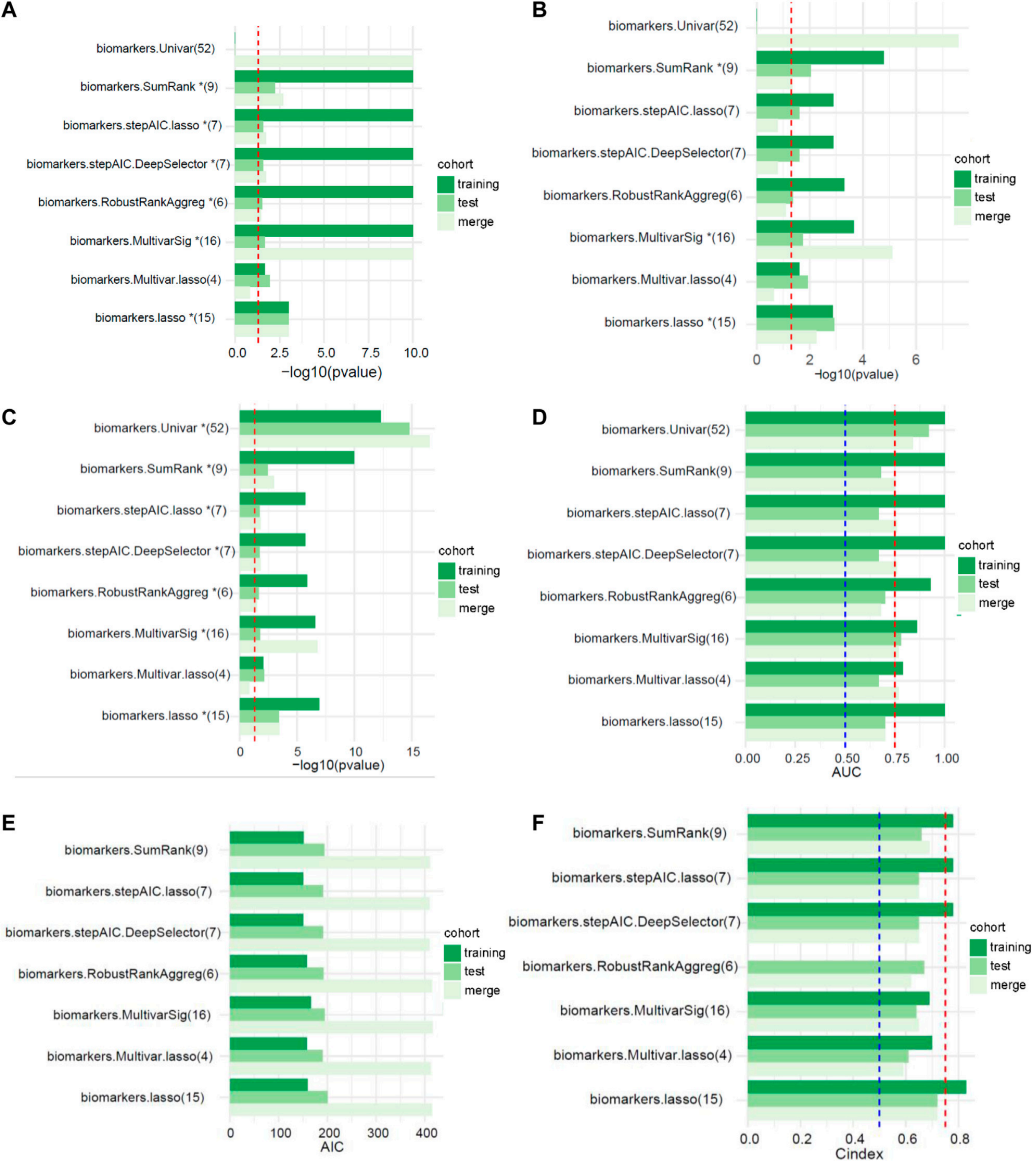
The performances of different signatures selected using multiple feature selection methods (the numbers in parentheses represent the number of lncRNAs in the signature). **(A)** Univariate analysis (the red dotted line represents *p* < 0.05). **(B)** Multivariate analysis (the red dotted line represents *p* < 0.05). **(C)** Kaplan-Meier survival curve (the red dotted line represents *p* < 0.05). **(D)** AUC values of the ROC curves (the red and blue dotted lines represent 0.75 and 0.5, respectively). **(E)** Akaike’s information criterion (AIC). **(F)** C-index of the logistic regression model (the red and blue dotted lines represent 0.75 and 0.5, respectively).

The workflow for this study is shown in Figure 1. The TCGA dataset containing both mRNA and lncRNA expression data was considered, and the resulting 354 GC patients were first randomly divided into training and test cohorts at a ratio of 1:1. After preprocessing from TCGA database, 250 patients with GC (123 in the training cohort and 127 in the test cohort) with complete information on survival time, survival status, and TNM stage were selected for the survival analysis (Supplementary Table S1). The training cohort was used to establish the risk model. We performed a comprehensive analysis named TCGA Biomarkers to identify prognosis-related lncRNA signatures (Figure 2). Univariate Cox regression analyses, multivariate Cox regression analyses, Kaplan-Meier survival curve, AUC values of the ROC curves, and AIC values were applied to assess the quality of various lncRNA combination models and further optimize the lncRNA risk model. Finally, we found that the risk model selected using the SumRank method was the best, which comprised MIR1.1HG, LOH12CR2, LINC02975, LOC100506405, ERCC8.AS1, LINC02763, LINC02985, LINC00520, and LINC00567 (Supplementary Figure S1A).

### Establishment and validation of the risk model

**FIGURE 3:**
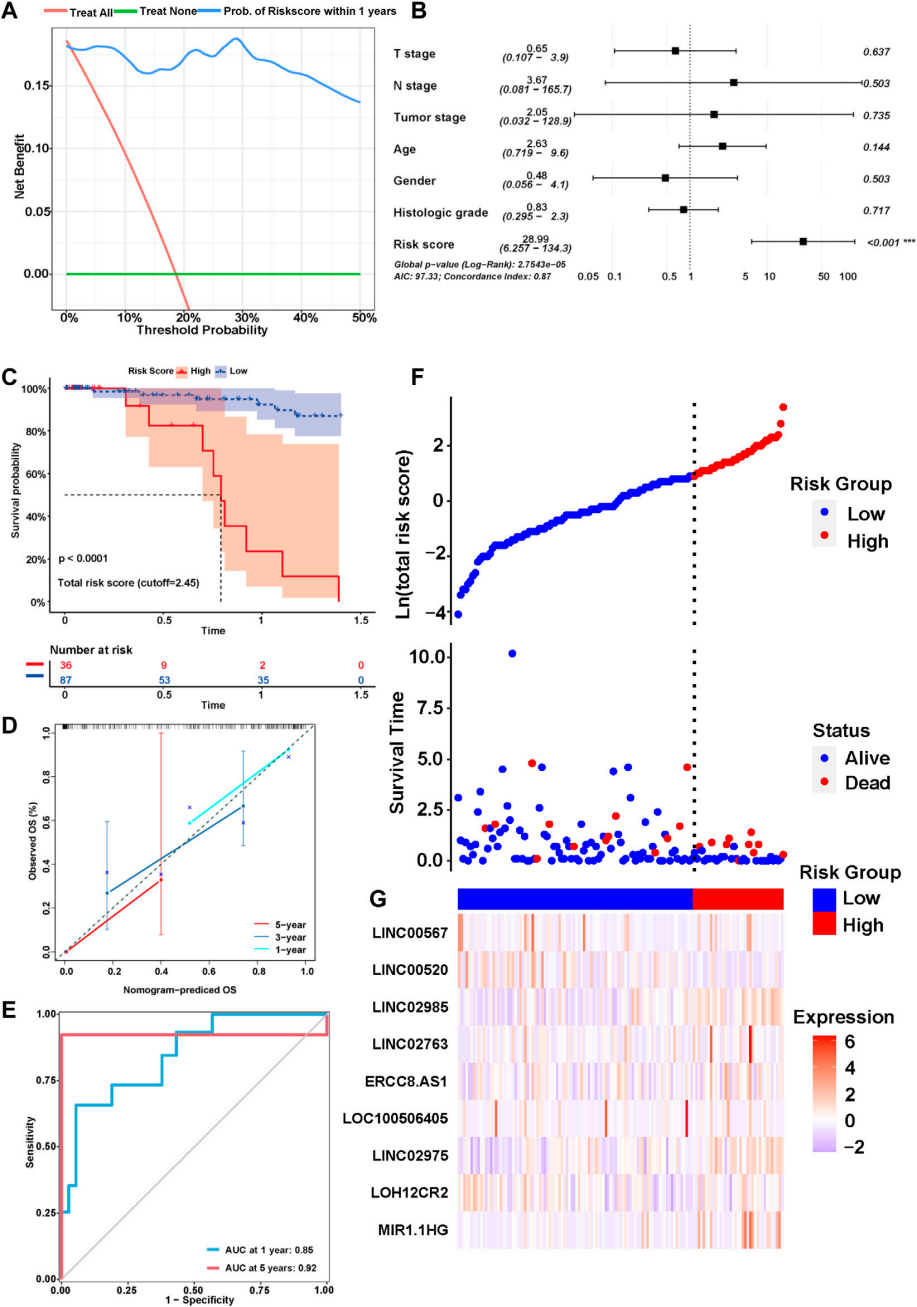
Identification of the prognostic signature in the training cohort. **(A)** Decision curve analysis (DCA) was conducted to confirm the superiority of the risk score. **(B)** Multivariable analysis was conducted to validate the independent prognosis value of the model in the training cohort. **(C)** Kaplan-MeierKaplan-Meier curves of the signature for predicting the overall survival (OS) of GC patients. **(D)** The calibration curves were constructed to determine the accuracy of the nomogram for OS at 1, 3 and 5 years. **(E)** Time-dependent ROC curve analysis of the risk model in different years. **(F,G)** The distribution of the expression of the nine lncRNAs in the high- and low-risk groups.

**FIGURE 4:**
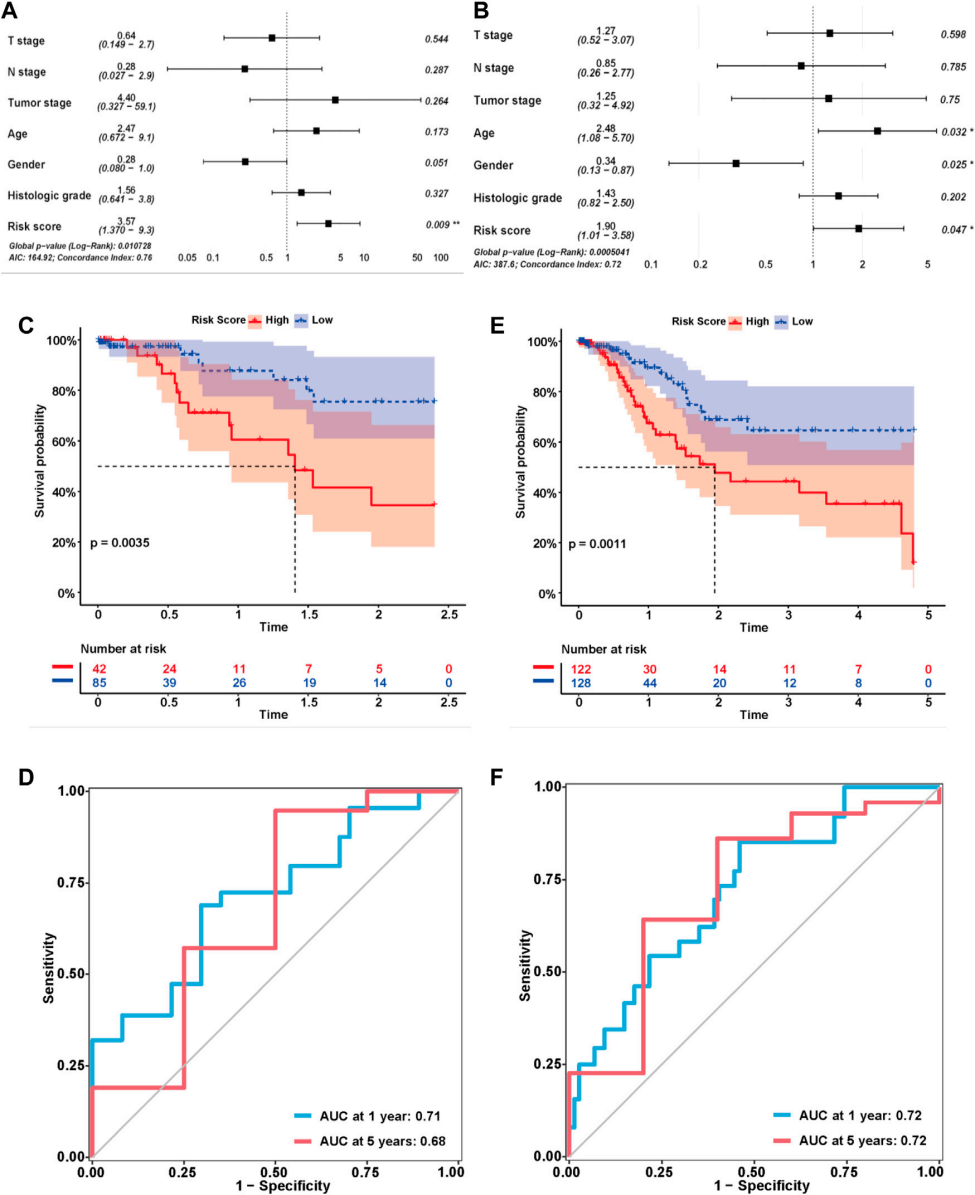
Validation of the risk model in the test cohort and merged cohort. **(A,B)** Multivariable analysis was conducted to validate the independent prognostic value of the model in the test and merged cohorts. **(C,E)** Kaplan-MeierKaplan-Meier curves of the lncRNA signature for predicting OS in the test and merged cohorts. **(D,F)** Time-dependent ROC curve analysis of the risk model in the test and merged cohorts.

We explored the correlation between the risk score and clinical characteristics of the patients in the training cohort (Supplementary Table S2) dividing the patients into high- and low-risk groups according to the best ROC curve. DCA was performed, and the results revealed that the risk model had a higher efficacy (Figure 3A). The multivariate regression analysis suggested that the risk score was useful as an independent prognostic index (Figure 3B). The Kaplan‒Meier survival curves showed that the survival outcomes of patients in the high-risk group were significantly worse than those in the low-risk group (*p* < 0.0001) (Figure 3C). The calibration curves were subsequently plotted to determine the accuracy of the nomogram for OS at 1, 3 and 5 years (Figure 3D). We performed a ROC curve analysis of different years to further validate the accuracy of the risk model in predicting the survival outcome of patients with GC and found that the lncRNA signature potentially represented a prognostic marker for patients with GC. The AUCs obtained from the model at 1 and 5 years were 0.85 and 0.92, respectively, indicating that the risk model had sufficient efficacy (Figure 3E). In addition, more patients died in the high-risk group than in the low-risk group (Figure 3F). The expression levels of the nine lncRNAs in the model were visualized using a heatmap (Figure 3G). The formula and strategies applied in the training cohort were applied independently in the test cohort and merged dataset to evaluate the robustness of the lncRNA signature in predicting the prognosis. The characteristics of the patients in the two cohorts are shown in Supplementary Tables S3, S4. The risk score was also proven to be an independent risk factor in the validation group and the merged group in multivariate regression analysis (Figure 4A,B). Patients in the high-risk groups also revealed significantly worse OS than those in the low-risk groups in the test cohort (*p* = 0.0035) and merged dataset (*p* = 0.0011) (Figure 4C,E). Then, we used time-dependent ROC curves to test the accuracy of the risk model in both cohorts and observed that our risk model performed well in both groups (Figure 4D,F). Based on these results, this risk model was effective at predicting survival outcomes of patients with GC.

### Correlations between the risk score and clinicopathological characteristics

We also explored the relationship between the risk score and clinicopathological characteristics of GC patients in the training, test and merged cohorts (Table 1). In the training cohort, the patients with high-risk scores were significantly associated with more advanced histologic grade (*p* = 0.001), higher ESTIMATE scores (*p* < 0.001), higher immune (*p* = 0.022) and stromal (*p* < 0.001) scores and a worse prognosis (*p* = 0.012) than those with low-risk scores. In the test cohort, the patients with high-risk scores were significantly associated with a more advanced TNM stage (*p* = 0.035), higher ESTIMATE scores (*p* = 0.013) and immune scores and a worse prognosis (*p* < 0.001) than those with low-risk scores. As the number of patients increased, the patients with high-risk scores in the merged cohort had a more advanced histologic grade (*p* = 0.014) and TNM stage (*p* = 0.027), higher ESTIMATE scores (*p* < 0.001), higher immune (*p* < 0.001) and stromal (*p* = 0.001) scores and a worse prognosis (*p* = 0.009) than those with low-risk scores. As previously reported, favorable OS was observed for patients with high stromal scores and immune scores using the stromal-immune score-based gene signature (Wang et al., 2019). The histologic grade and TNM stage are commonly used as clinical indicators of the prognosis of patients with GC. These results indicated that the risk model was related to the immune status of patients and might be used as a preferred prognostic biomarker in all patients.

**TABLE 1:** Correlations between risk score and clinicopathologic characteristics of GC patients.

Characteristic	Risk score (training cohort, n = 123)			Risk score (test cohort, n = 127)			Risk score (merge cohort, n = 250)		
	High (%) *n* = 36	Low (%) *n* = 87	*p*-value	High (%) *n* = 42	Low (%) *n* = 85	*p*-value	High (%) *n* = 122	Low (%) *n* = 128	*p*-value
Age									
< 60 years	14 (39%)	30 (34%)	0.6	11 (26%)	26 (31%)	0.6	37 (30%)	44 (34%)	0.5
≥ 60 years	22 (61%)	57 (66%)		31 (74%)	59 (69%)		85 (70%)	84 (66%)	
**Gender**									
male	25 (69%)	59 (68%)	0.9	24 (57%)	51 (60%)	0.8	82 (67%)	77 (60%)	0.2
female	11 (31%)	28 (32%)		18 (43%)	34 (40%)		40 (33%)	51 (40%)	
**T stage**									
T 1–2	6 (17%)	26 (30%)	0.13	7 (17%)	23 (27%)	0.2	25 (20%)	37 (29%)	0.12
T 3–4	30 (83%)	61 (70%)		35 (83%)	62 (73%)		97 (80%)	91 (71%)	
**N stage**									
N0	14 (39%)	27 (31%)	0.4	6 (14%)	23 (27%)	0.11	29 (24%)	41 (32%)	0.15
N1-3	22 (61%)	60 (69%)		36 (86%)	62 (73%)		93 (76%)	87 (68%)	
**Tumor stage**									
IA-IIA	11 (31%)	25 (29%)	0.8	6 (14%)	27 (32%)	0.035	25 (20%)	44 (34%)	0.014
IIB-IV	25 (69%)	62 (71%)		36 (86%)	58 (68%)		97 (80%)	84 (66%)	
**Histologic grade**									
G1	0 (0%)	3 (3.4%)	0.001	1 (2.4%)	0 (0%)	0.2	2 (1.6%)	2 (1.6%)	0.027
G2	6 (17%)	41 (47%)		10 (24%)	28 (33%)		32 (26%)	53 (41%)	
G3	30 (83%)	43 (49%)		31 (74%)	57 (67%)		88 (72%)	73 (57%)	
**Estimate score**	2,065 (1,312, 2,886)	821 (−279, 2,041)	<0.001	1,583 (817, 2,756)	946 (−62, 1,964)	0.013	1,627 (817, 2,727)	754 (−176, 1,897)	<0.001
**Immune score**	1,481 (946, 1,985)	912 (365, 1,588)	0.022	1,520 (730, 1,815)	986 (407, 1,467)	0.010	1,455 (664, 1,867)	831 (267, 1,387)	<0.001
**Stromal score**	757 (160, 1,008)	−38 (−688, 341)	<0.001	403 (−202, 842)	51 (−556, 519)	0.055	271 (-208, 866)	−34 (−659, 518)	0.001
**Status**	9 (25%)	6 (6.9%)	0.012	14 (33%)	8 (9.4%)	<0.001	31 (25%)	16 (12%)	0.009

### Clinical significance of the risk model

After confirming the relationship between the risk score and prognosis, we explored the clinical importance of the risk model in the merged cohort. The association of risk scores with clinicopathological characteristics of patients with GC was assessed by comparing the score distributions among different age, gender, T stage, N stage, tumor stage, histologic grade, and survival status groups (Supplementary Figure S1C–H). Patients with GC presenting an advanced TNM stage (*p* = 0.0047) and histologic grade (*p* = 0.0073) had significantly higher risk scores. Additionally, the surviving patients had lower risk scores than the dead patients (*p* = 0.0011). Therefore, the risk score calculated using our risk model indeed served as a reliable prognostic predictor in patients with GC.

### Performance of the selection strategies

We randomly divided TCGA cohort at a 1:1 ratio with 1000 replicates to verify the performance of the 11 selection strategies We compared the effectiveness of different methods for predicting patient outcomes. Most of the risk models screened using the univariate method were composed of more than 50 lncRNAs, which lacked accuracy. Therefore, this method was not considered in the performance comparison. SumRank was more effective at predicting the prognosis than the other methods except the univariate method (Supplementary Figure S2A,B). Then we also compared other metrics of model performance, such as AIC, C-index and *p* value of the multivariate Cox regression analysis of the risk models in the training, test and merged cohorts. The SumRank method had the most stable performance, consistently ranking at the top (Supplementary Figure S2C–H).

### Functional enrichment analysis in the merged cohort

**FIGURE 5:**
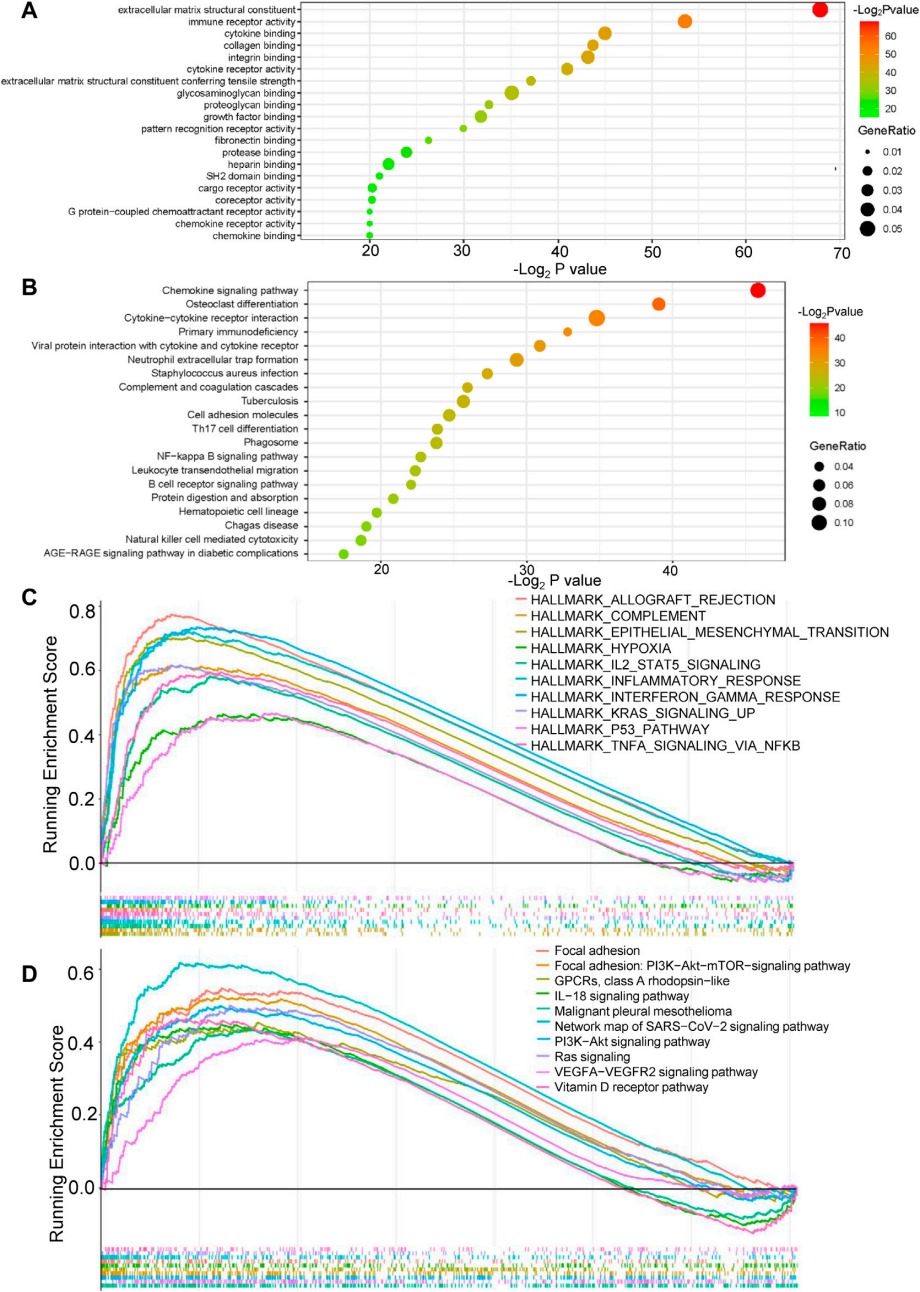
Functional enrichment analysis of the risk model. **(A,B)** Gene Ontology (GO) annotations and Kyoto Encyclopedia of Genes and Genomes (KEGG) enrichment analysis. **(C)** Gene set enrichment analysis (GSEA) in the MSigDB database of tumor hallmarks. **(D)** WikiPathways analysis.

**FIGURE 6:**
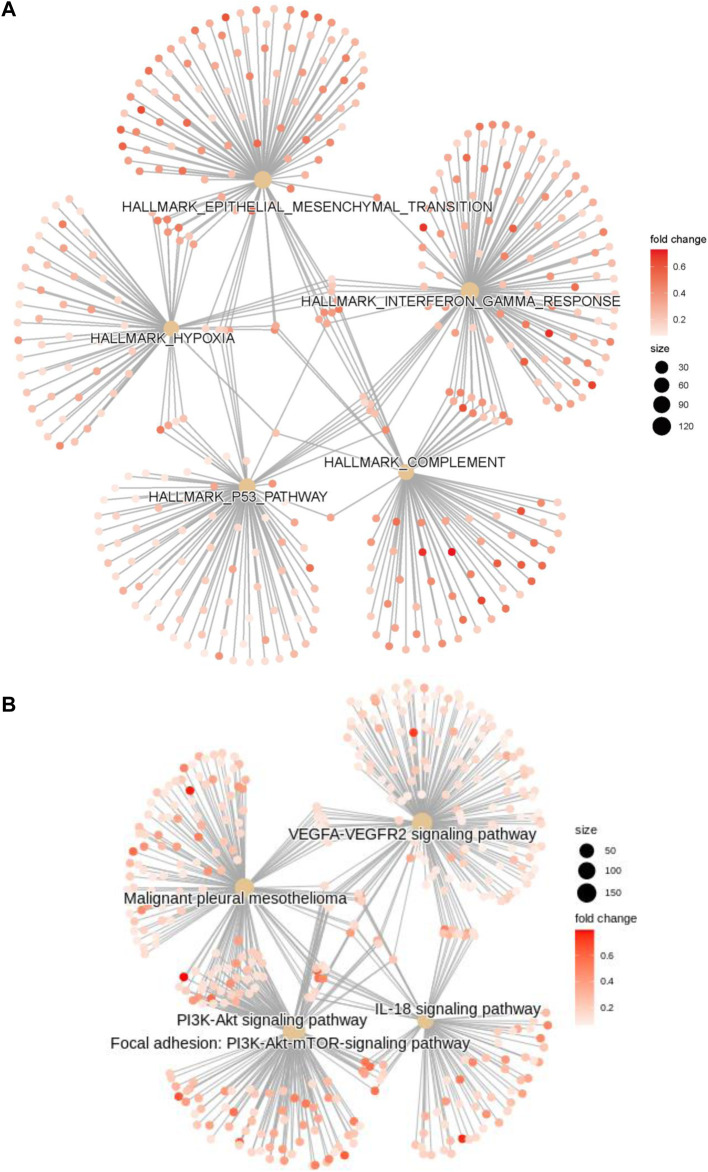
The cnetplots show the relationship between tumor hallmarks and metabolic pathways **(A,B)**.

GO, KEGG, GSEA, and WikiPathways analyses were performed using gene expression data from TCGA cohort to better understand the molecular processes occurring in the high-risk and low-risk groups. GO annotations and KEGG enrichment analysis showed that the DEGs in the high-risk group were mainly enriched in immune-related pathways such as “immune receptor activity”, “cytokine binding”, “cytokine receptor activity”, “chemokine signaling pathway”, “cytokine-cytokine receptor interaction”, “primary immunodeficiency”, “Th17-cell differentiation”, “B-cell receptor signaling pathway” and “natural killer cell mediated cytotoxicity”, and unlike those in the low-risk group (Figure 5A,B, Supplementary Tables S5 ,S6). In addition, many cancer-related processes were enriched, such as “integrin binding”, “fibronectin binding”, “complement and coagulation cascades”, “cell adhesion molecules” and “NF-kappa B signaling pathway”. GSEA of tumor hallmarks in the MSigDB database revealed that the high-risk group also showed an enrichment in common signaling pathways, including “epithelial-mesenchymal transition (EMT)”, “hypoxia”, “KRAS signaling”, and “p53 pathway”, and immune-related pathways, including “IL-2-STAT5 signaling”, “inflammatory response”, “interferon gamma response”, and “TNF-α signaling *via* NF-κB” (Figure 5C and Supplementary Table S7). The WikiPathways analysis showed the enrichment of metabolic pathways, including “focal adhesion”, “PI3K-Akt-mTOR signaling pathway”, “Ras signaling”, and “VEGFA-VEGFR2 signaling pathway” (Figure 5D and Supplementary Table S8). The cnetplots show the relationship between tumor hallmarks and metabolic pathways (Figure 6A,B). The difference in the enrichment of nine cancer-related pathways between the low- and high-risk groups was also assessed (Supplementary Figure S3A–I). These results indicated that the high-risk group exhibited significant enrichment of signaling pathways associated with cancer development and tumor immunity. This finding provides further understanding of the functional roles and immunomodulatory mechanisms of the lncRNA signature.

### Correlations between the risk score, immune cell infiltration, and immunotherapy efficacy

**FIGURE 7:**
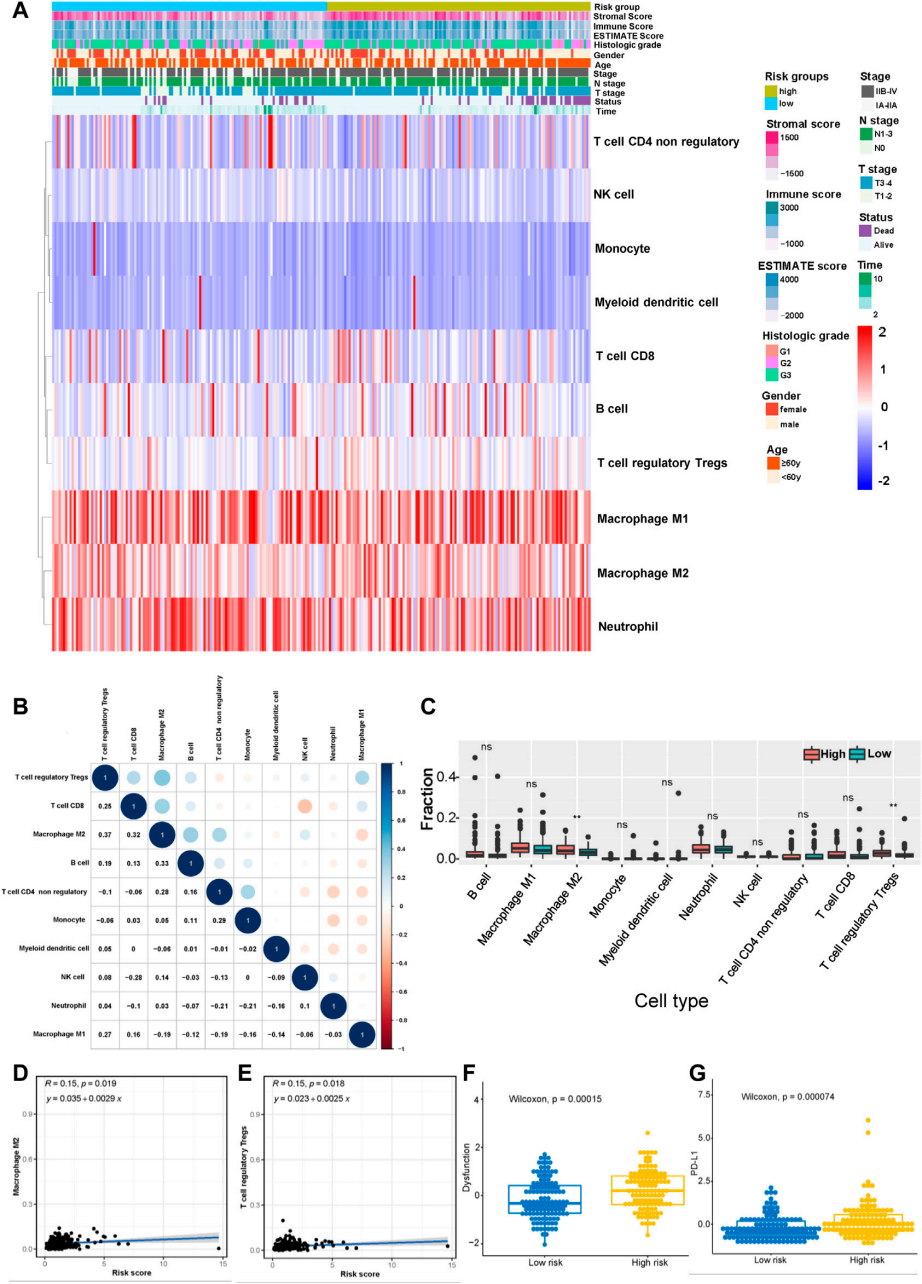
Correlation between the risk model and the immune status of the tumor microenvironment (TME). **(A)** Heatmap showing the distribution of clinical features and immune cell infiltration. **(B)** Correlation between 10 tumor infiltrating immune cell types (red represents a negative correlation between two immune cell types and blue represents a positive correlation between two immune cell types; the larger the shape of the point, the stronger the correlation). **(C)** The distribution of 10 tumor infiltrating immune cell types in the high- and low-risk groups (**p < 0.01). Correlation between the risk score and infiltrating levels of M2-type macrophages **(D)** and Tregs **(E)**. **(F)** The T-cell dysfunction scores in the high-risk and low-risk groups calculated using the TIDE algorithm. **(G)** The expression of PD-L1 in the high- and low-risk groups.

According to the results described above, the risk model was closely related to immune pathways. Then, we explored the relationship between this model and immune cell infiltration and immunotherapy efficacy based on the gene expression data obtained from the merged cohort. The immune cell infiltration status was calculated by performing a quanTIseq analysis (Supplementary Table S9). Figure 7A shows the infiltration of 10 main immune cell types in different risk score groups. Figure 7B shows the correlation among 10 immune cell types, and the results indicated that the levels of immunosuppressive M2-type macrophages had a positive correlation with the levels of Tregs and a negative correlation with the levels of M1-type macrophages. Then, we measured the levels of infiltration of 10 immune cell types in the high-risk group and the low-risk group, and the results showed that the levels of M2-type macrophage and Treg infiltration in the high-risk group were much higher than those in the low-risk group (Figure 7C). The correlation between the levels of these two immune cell types and the risk score was measured, and the results revealed that the levels of M2-type macrophages and Tregs correlated positively with the risk score (Figure 7D,E). Based on these results, the status of the tumor immune microenvironment was immunosuppressive in the high-risk group unlike in the low-risk group, which was closely related to the effect of tumor immunotherapy. Furthermore, we measured the T-cell dysfunction scores in the high-risk and low-risk groups using the TIDE algorithm and found that the high-risk group exhibited higher levels of T-cell dysfunction (*p* = 0.00015, Figure 7F). In addition, PD-L1 was expressed at much higher levels in the high-risk group was than in the low-risk group (*p* = 0.000074, Figure 7G), which indicated a potentially better anti-PD-1/PD-L1 therapeutic response in the high-risk group.

### Correlation between the risk model and tumor mutation burden

**FIGURE 8:**
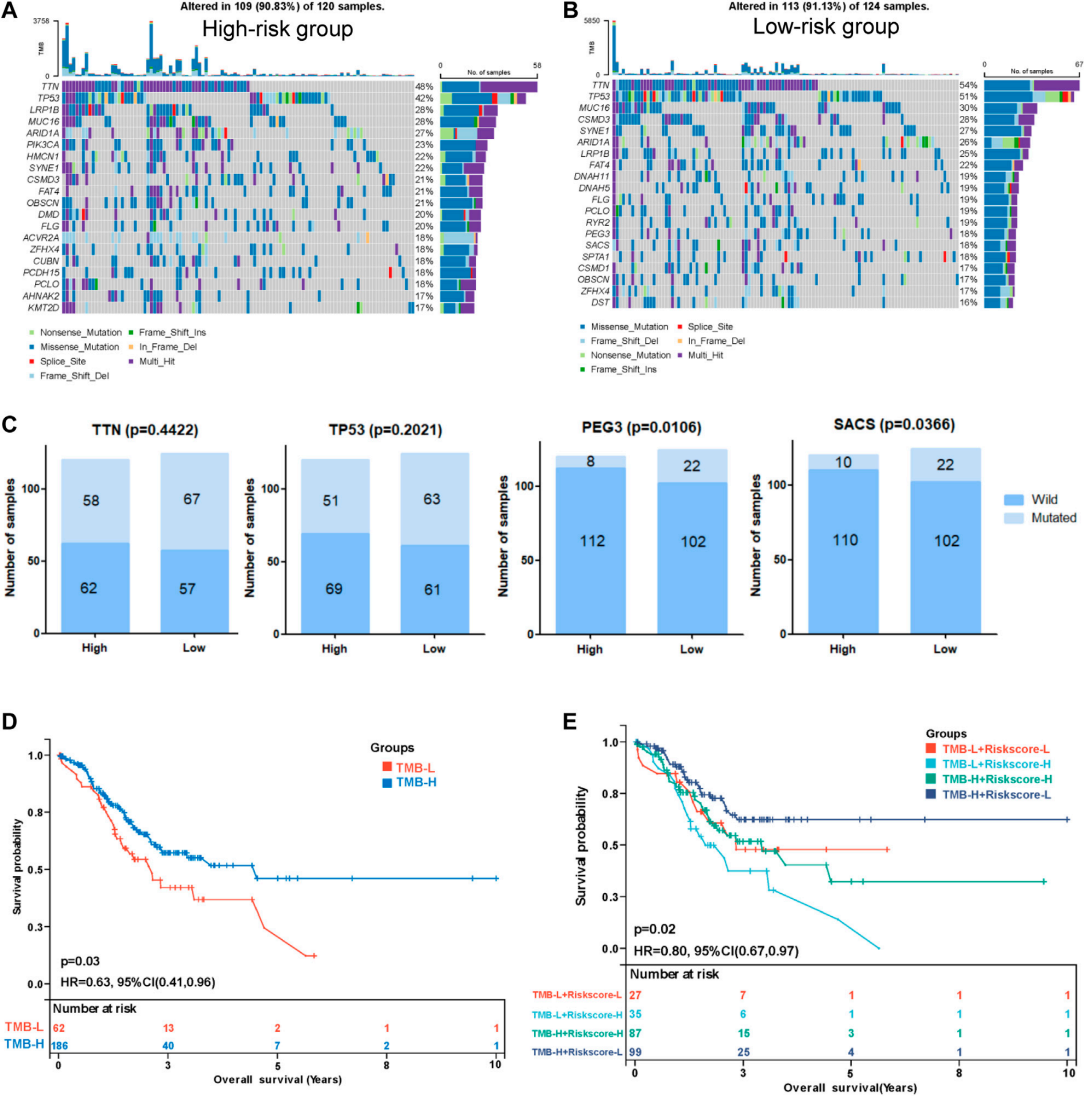
Correlation between the risk score and tumor mutation burden (TMB). Waterfall plot displaying the distribution of the top 20 mutated genes with the highest mutation frequency in the high- **(A)** and low-risk **(B)** groups. **(C)** Comparisons of the mutation status of TTN, TP53, PEG3, and SACS. **(D)** Kaplan-Meier curve analysis of the OS between the high-TMB group and the low-TMB group. **(E)** Kaplan-Meier curve analysis of OS of patients with low- or high-risk scores in the high-TMB group and low-TMB group.

We performed a tumor mutation analysis to assess the difference in the TMB between the high- and low-risk groups using “maftools” in R (Supplementary Tables S10, S11. The waterfall plot showed the distribution of the top 20 mutated genes with the highest mutation frequency in the two groups (Figure 8A,B). TTN and TP53 were the genes with the highest mutation frequency in the two groups of patients, and the low-risk group exhibited a trend toward a higher mutation frequency than the high-risk group, but the difference was not significant (Figure 8C). Other commonly mutated genes, such as PEG3 and SACS, were significantly more frequently mutated in the low-risk group than in the high-risk group. We subsequently analyzed the relationship between the TMB and the prognosis of patients and found that patients with a lower TMB had poorer survival outcomes (Figure 8D). Then, we explored the relationship between the TMB combined with the risk score and prognosis. Intriguingly, TMB-high patients with low-risk scores had the best survival outcomes, and TMB-low patients with high-risk scores had the worst survival outcomes (Figure 8E), indicating that the TMB combined with the risk model might be applicable for predicting the prognosis.

### External validation and evaluation of the expression of nine lncRNAs

**FIGURE 9:**
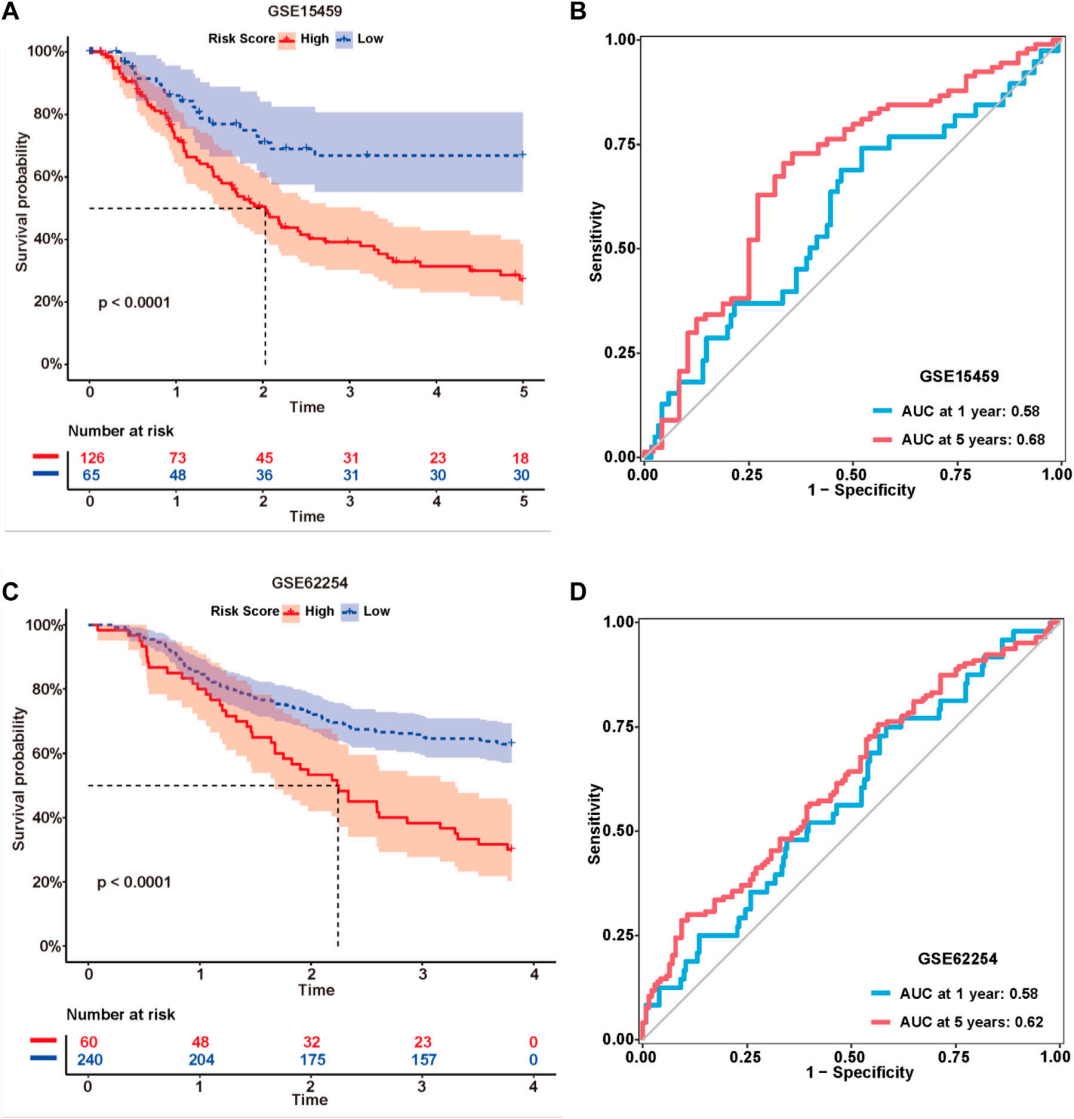
External validation of the risk model. Kaplan-Meier curves of the lncRNA signature for predicting the OS of patients in the GSE15459 **(A)** and GSE62254 **(C)** datasets. Time-dependent ROC curves of the signature in the GSE15459 **(B)** and GSE62254 **(D)** datasets.

**FIGURE 10:**
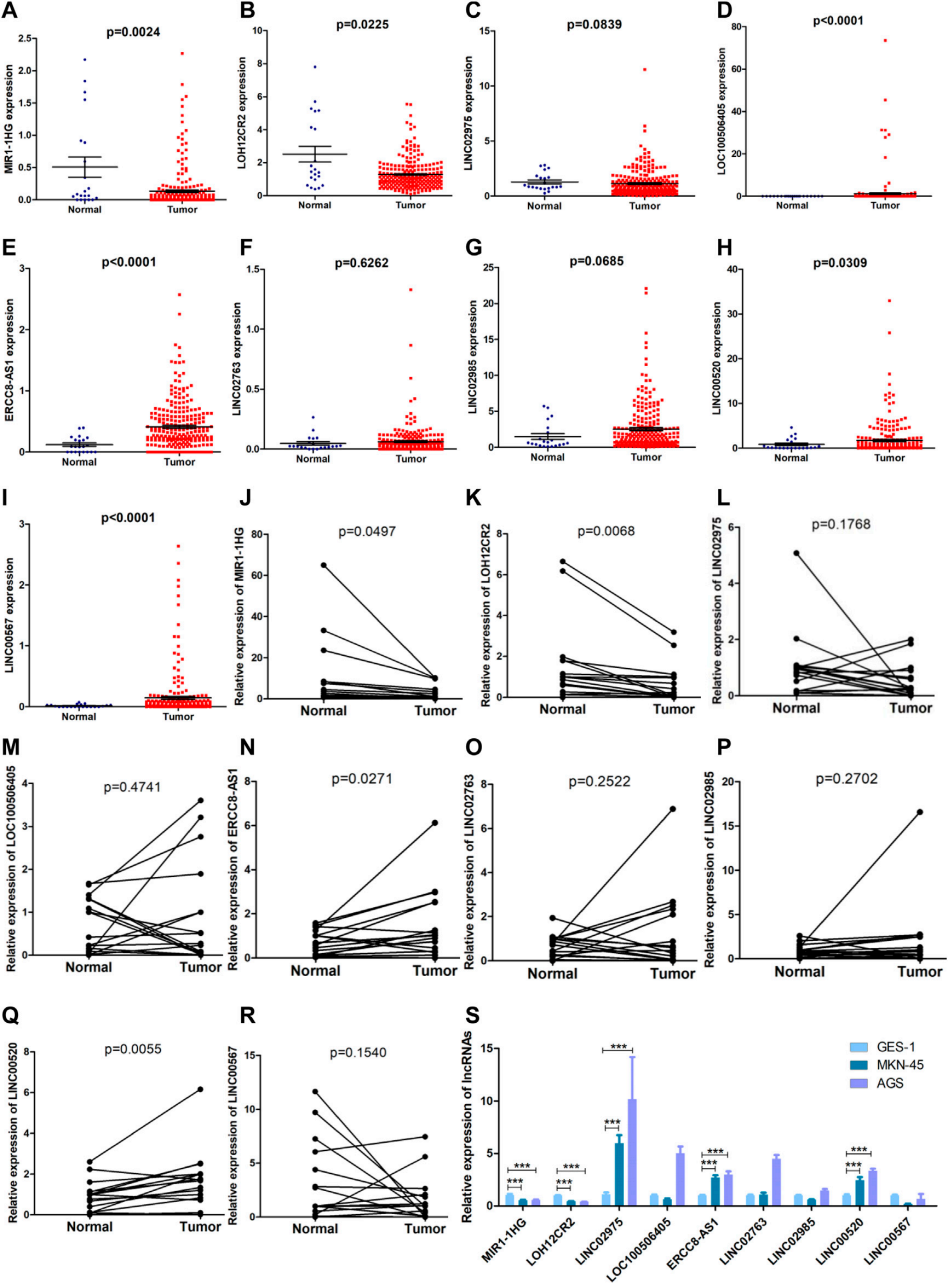
Evaluation of the expression of nine lncRNAs in the risk model. **(A–I)** Comparison of the expression levels of the nine lncRNAs between tumor tissues and normal tissues in TCGA datasets (Mann-Whitney *U* test). **(J–R)** The expression levels of the nine lncRNAs in 16 paired GC samples detected using qRT-PCR (paired *t* test). **(S)** The expression levels of the nine lncRNAs in two human gastric cancer cell lines (MKN-45 and AGS) and one human gastric epithelial cell (GES-1) (Mann-Whitney *U* test, ***: *p* < 0.001).

The formula used in the training cohort from TCGA datasets was applied independently to patients in two GEO datasets (GSE15459 and GSE62254) to further verify the effectiveness of the model. As expected, patients in the high-risk group had a worse prognosis than those in the low-risk group in both GEO datasets (*p* < 0.0001) (Figure 9A,C). The results from the time-dependent ROC curves showed that the 5-year average AUC values were 0.68 and 0.62 (Figure 9B,D), respectively, indicating that the risk model performed well in both datasets. Thus, the nine lncRNAs in our risk model were associated with the survival outcomes of patients with GC. Then, we evaluated the expression levels of these lncRNAs in TCGA datasets, tissues and cell lines. Six of nine lncRNAs (MIR1.1HG, LOH12CR2, LOC100506405, ERCC8.AS1, LINC00520, and LINC00567) were differentially expressed between tumor tissues and normal tissues in TCGA datasets (Figures 10A–I). We also collected 16 paired GC samples and validated the expression levels of these nine lncRNAs using qRT-PCR (Figure 10J–R). All primers used in the qRT-PCR assay are listed in Supplementary Table S12. MIR1.1HG and LOH12CR2 were expressed at higher levels in normal tissues than in cancer tissues, while the expression levels of ERCC8.AS1 and LINC00520 in tumor tissues were significantly higher than those in paired adjacent normal tissues, which showed similar expression trends to TCGA datasets. In addition, we tested the expression levels in two human gastric cancer cell lines (MKN-45 and AGS) and one human gastric epithelial cell line (GES-1). Similar trends for the expression of MIR1.1HG, LOH12CR2, and ERCC8.AS1 and LINC00520 were observed (Figure 10S). Interestingly, we also observed that LINC02975 was expressed at significantly higher levels in cancer cell lines than in the gastric epithelial cell line, while only a similar expression trend was observed in TCGA dataset, but the *p* value was not statistically significant. Based on these results, MIR1.1HG, LOH12CR2, ERCC8.AS1, and LINC00520 might exert vital functions in the development of GC.

## Discussion

GC is one of the most common malignant tumors of the digestive system and has a poor prognosis, despite the improvements in treatment strategies. Due to the heterogeneity of GC, developing a prognostic assessment system to guide individualized clinical treatment remains a challenge. Numerous studies have shown that the interaction between tumor cells and immune cells in the TME plays crucial roles in the occurrence and development of GC, immune escape and chemotherapy resistance (Rojas et al., 2020). As important immune regulators, lncRNAs might serve as potential biomarkers and therapeutic targets. Several previous studies have generated different lncRNA signatures to predict survival and immune features in GC (Liang et al., 2021; Ma et al., 2021; Xin et al., 2021; Nie et al., 2022; Wang et al., 2022), but these studies still have some issues that should be discussed. For example, the clinical data obtained from the databases should be preprocessed and standardized since some studies utilize TNM staging methods based on very old versions of the standards; patients with distant metastases should be excluded from the analysis since these data have a significant impact on the prognostic prediction. These studies often generated the signatures using various feature selection methods, but the authors did not clearly detemine which one was more advantageous (Li et al., 2020). In the present study, we developed an integrative pipeline to construct a prognosis-related lncRNA signature using a machine learning algorithm and verified the performance of the 11 selection strategies for 1000 times randomly. The advantage of integrative procedures is that they fit a model with consensus prognostic performance based on multiple feature selection methods using a machine learning algorithm, which may further reduce the dimension of variables and simplify the model to facilitate its translational (Liu Z. et al., 2022). We developed a process for the first time to verify the performance of the different selection strategies and compared multiple metrics of model performance, such as AIC, C-index and *p value* of multivariate a Cox regression analysis of the risk models, which would provide a reference for the evaluation of model effectiveness. Additionally, some lncRNA models reported previously lacked the necessary validation in independent cohorts. Therefore, prognostic markers of GC must be established by screening suitable methods for generating models and standardizing clinical data.

In this study, clinical data were first standardized and screened, and patients were divided into the independent training set and validation set at a 1:1 ratio. Then, we eliminated the data obtained from unsuitable patients for analysis. First, we used machine learning to develop a risk score and integrated 11 feature selection methods, including univariate and multivariate Cox regression, LASSO, stepAIC, subset selection, the RobustRankAggreg method, the SumRank method and a combination of the above methods, using data obtained from the training cohort. Then, we found that the risk model selected by the SumRank method was the best according to the univariate Cox regression analysis, multivariate Cox regression analysis, Kaplan-Meier survival curve, AUC values of the ROC curves and AIC. Nine prognosis-related lncRNAs (MIR1.1HG, LOH12CR2, LINC02975, LOC100506405, ERCC8.AS1, LINC02763, LINC02985, LINC00520, and LINC00567) were selected to establish the risk model. Among these lncRNAs, LOH12CR2, ERCC8.AS1, LINC02985, LINC00520, and LINC00567 have been previously demonstrated to exert crucial roles in the progression of various tumors, including GC, while others were reported for the first time (Luan et al., 2020; Lina, 2021; Rothzerg et al., 2021; Tseng et al., 2021; Liu B. et al., 2022). To test the efficacy of the model, we divided patients into a high-risk group and a low-risk group according to the ROC curve. Then, we performed a survival analysis and found that patients in the low-risk group had better survival outcomes than the high-risk group of patients in the training cohort. DCA and ROC curve analysis were conducted to validate the efficacy and accuracy of the risk model. The results demonstrated that the risk model could be used as a promising prognostic biomarker in GC patients. The selection methods used in the training cohort were applied independently to the test and merged datasets and two external GEO datasets to verify the stability of the risk model in the prognostic prediction. The results showed that the risk model was effective at predicting survival outcomes. We also explored the correlations between the risk score and clinicopathological characteristics in the three cohorts and found positive correlations between the risk score and histologic grade, clinical stage, ESTIMATE score, immune score and stromal score, indicating the essential roles of the components of the lncRNA signature in GC progression. The performance of the risk model improved as the number of patients in the cohort increased. These results strongly suggested that our risk model could be used as a stable predictor of prognosis in GC patients. This result also suggested that our model building method was particularly suitable for diseases with few databases.

Numerous studies have shown that immune checkpoints and the immune cell infiltration status are important factors affecting the prognosis and immunotherapy effectiveness of cancers, including GC (Kono et al., 2020; Puliga et al., 2021; Huang et al., 2022b). We performed functional enrichment analyses of the merged cohort to better understand the immunomodulatory mechanisms of the components of the lncRNA signature. Several established cancer-related pathways and hallmarks, including the “NF-kappa B signaling pathway”, “EMT”, “hypoxia”, “KRAS signaling”, and “p53 pathway”, were enriched in the high-risk group, indicating the internal regulatory mechanisms of the components of the lncRNA signature in the progression and metastasis of GC. We also observed that the terms correlated with many immune-related pathways, such as “cytokine-cytokine receptor interaction”, “primary immunodeficiency”, “Th17-cell differentiation”, “B-cell receptor signaling pathway”, and “natural killer cell mediated cytotoxicity”. In addition, several metabolic pathways, including “focal adhesion”, “PI3K-Akt-mTOR signaling pathway”, “Ras signaling”, and “VEGFA-VEGFR2 signaling pathway”, were also enriched in the high-risk group. Many of these signaling pathways have been reported to play an important role in regulating the remodeling of the tumor immune microenvironment (Zhou et al., 2019; Secker and Harvey, 2021). Then, we explored the relationship between the risk model, immune cell infiltration and immunotherapy efficacy. We found that the TME in the high-risk group was immunosuppressive, with a large number of infiltrating M2-type macrophages and Tregs. Additionally, TIDE analysis showed a higher incidence of T-cell dysfunction and higher expression of PD-L1 in the high-risk group. Many studies have revealed that M2-type macrophages suppress the cell-mediated immune response and induce an immunosuppressive TME by recruiting Tregs. Therefore, these lncRNAs may affect tumor growth, metastasis and the response to immunotherapy by regulating the status of the tumor immune microenvironment. Recent studies have demonstrated that the TMB is emerging as a predictive biomarker for the response to immune checkpoint blockade (Chan et al., 2019; Jardim et al., 2021). We assessed the correlation between the risk score and TMB and found that many common mutated genes were significantly more frequently mutated in the low-risk group than in the high-risk group. Additionally, TMB-high patients with low-risk scores had the best survival outcomes and TMB-low patients with high-risk scores had the worst survival outcomes, indicating that using the TMB level combined with the risk model might be a better approach for predicting the prognosis than the use of either of these indicators alone. Taken together, the data suggest that the lncRNAs identified in our risk model may affect the infiltration and differentiation of immune cells by participating in specific signaling pathways of GC, thus affecting the TME status and the responses to immunotherapy. We evaluated the expression levels of these nine lncRNAs in TCGA datasets, clinical tissues and cell lines to further verify the effectiveness of the model and identify the most valuable lncRNAs. The trends in the expression of four lncRNAs (MIR1.1HG, LOH12CR2, ERCC8.AS1, and LINC00520) in TCGA datasets, clinical specimens and cell lines were highly consistent, indicating that these four lncRNAs might exert a more important role in the development of GC. Future experiments should be performed to explore the biological functions of these lncRNAs.

In this study, we used machine learning to develop an integrative analysis for screening risk prediction models using multiple feature selection methods. Although there are many methods that can be used to generate risk models based on lncRNA expression, few studies have standardized the clinical data. Through this study, we proposed a new risk model prediction scheme and preliminarily verified its effectiveness and accuracy. After establishing the risk model using data from the training cohort, we further validated the model for predicting the prognosis in the test cohort, full dataset and two external GEO datasets. The risk model was also proven to exert a crucial function in predicting the immune cell infiltration and immunotherapeutic responses of GC patients. Despite the positive findings, our study still has several limitations. First, all data and specimens used in this study were obtained retrospectively, and a prospective multicenter cohort of GC patients undergoing immunotherapy is needed to validate the prognostic and predictive utility of this model. Second, we failed to obtain larger amounts of data regarding both mRNA and lncRNA expression and patient clinical data in other databases. Some clinical data and molecular traits were not adequate, which might affect the associations between the risk model and clinical variables. In addition, some lncRNAs were reported for the first time in this study, the roles of most lncRNAs in GC remain unknown, and the molecular mechanisms of these lncRNAs require further experimental verification *in vivo* and *in vitro*.

In summary, we generated a novel prognosis-related lncRNA signature for predicting the prognosis of GC patients. Our findings provide ideas for integrating multiple screening methods for risk modeling through machine learning.

## Data Availability

The datasets presented in this study can be found in online repositories. The names of the repository/repositories and accession number(s) can be found in the article/Supplementary Material.

## Supplementary material

Supplementary Figure S1
**(A)** The forestmap shows the nine lncRNAs identified in the risk model. **(B–H)** Clinical significance of the risk model in the merged cohort. **(B)** Age. **(C)** Gender. **(D)** T stage. **(E)** N stage. **(F)** Tumor stage. **(G)** Histologic grade. **(H)** Survival status.

Supplementary Figure S2 The performance of the 11 selection methods. **(A)** Number of tries with a *p* value < 0.05 in relation to prognosis among 1000 randomized tries in the training, test and merged cohorts. **(B)** Number of tries with *p* value < 0.05 in the training, test, and merged groups simultaneously associated with prognosis among 1000 randomized tries. The distribution of AIC of the 11 selection methods in the training **(C)**, test **(D)** and merged **(E)** cohorts. The distribution of C-index of the 11 selection methods in the training **(F)**, test **(G)** and merged **(H)** cohorts. The distribution of p values of multivariate Cox regression of the 11 selection methods in the training **(I)**, test **(J)**, and merged **(K)** cohorts.

Supplementary Figure S3
**(A–I)** The enrichment of nine cancer-related pathways between the low- and high-risk groups in the merged cohort.
