# Supplementary figures and images for: Machine learning-based identification of a novel prognosis-related long noncoding RNA signature for gastric cancer
Source: Front Cell Dev Biol. 2022 Nov 11;10:1017767. doi: 10.3389/fcell.2022.1017767 (PMC9691877; doi:10.3389/fcell.2022.1017767)

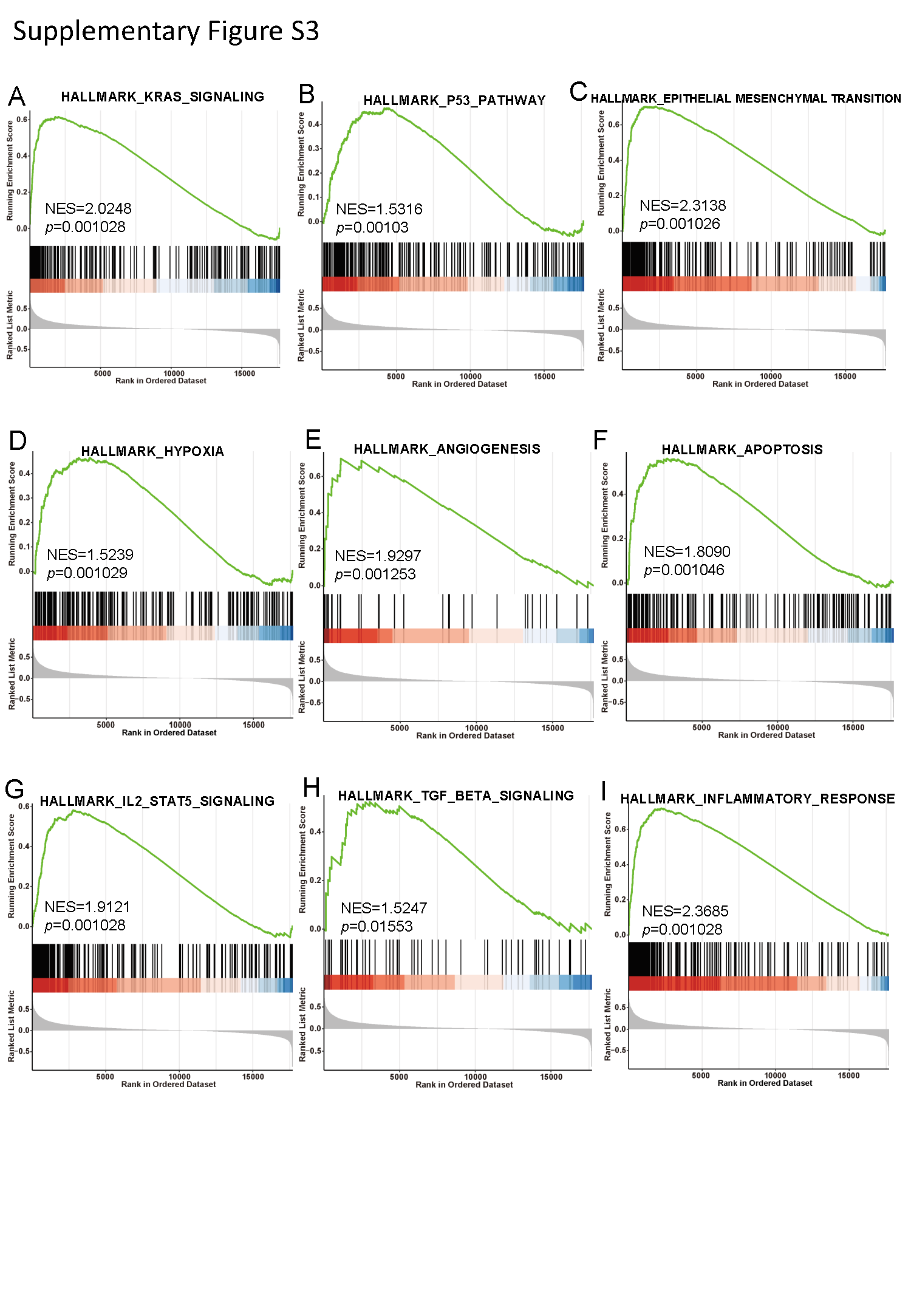

Supplement: Supplementary file 4 [file Image3.TIF]

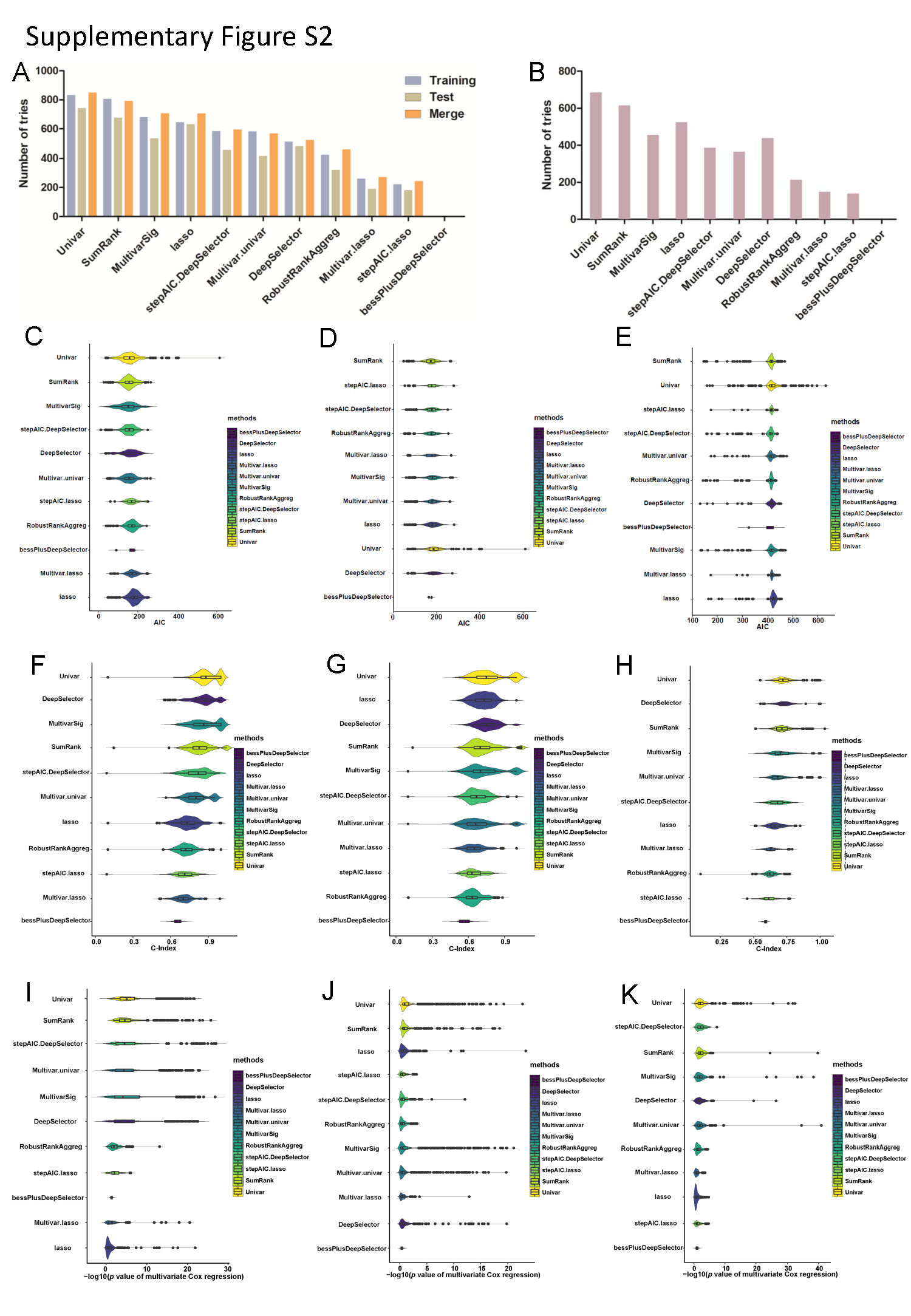

Supplement: Supplementary file 5 [file Image2.TIF]

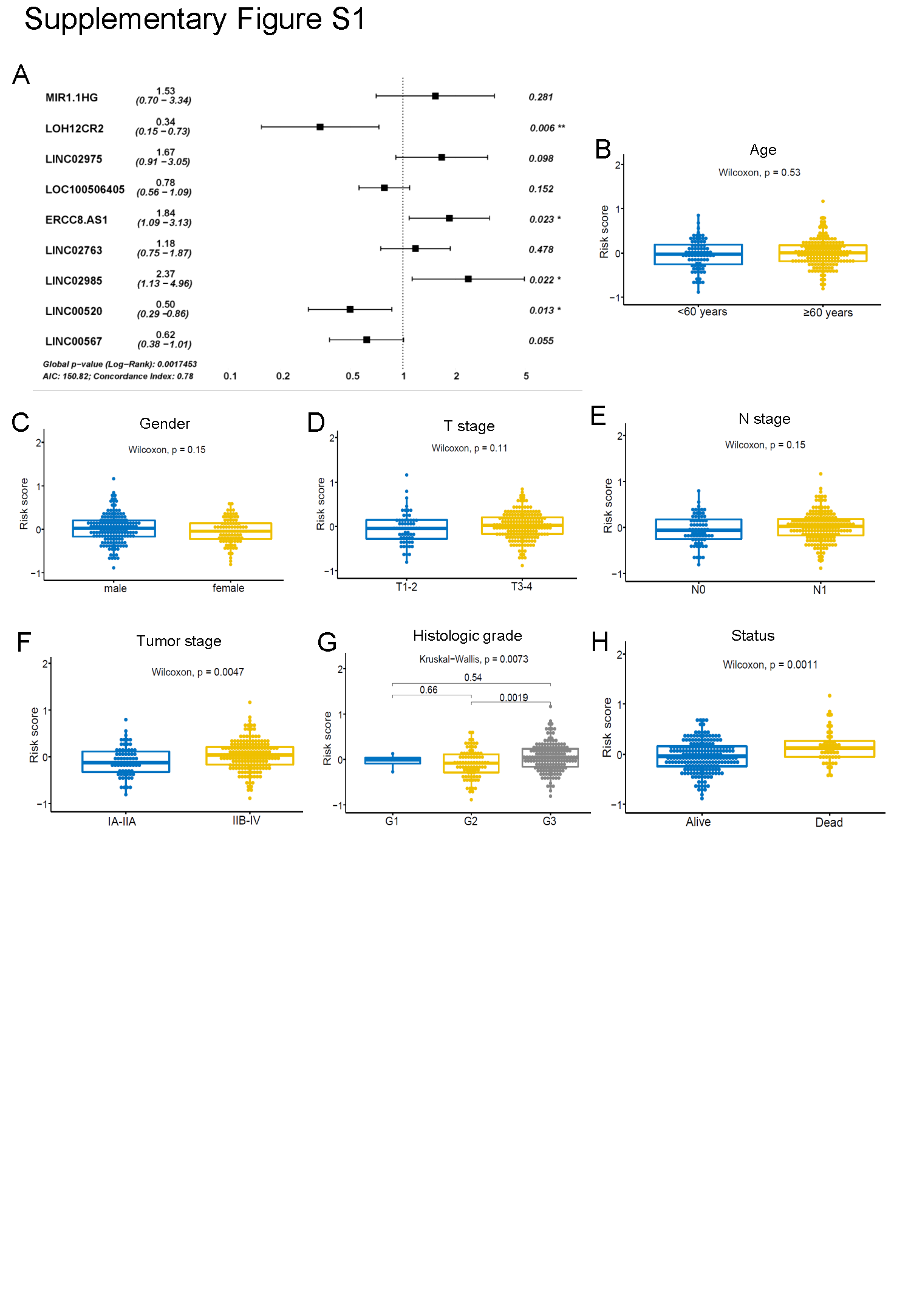

Supplement: Supplementary file 6 [file Image1.TIF]
